# Supplementary material for: The CTLH ubiquitin ligase substrates ZMYND19 and MKLN1 negatively regulate mTORC1 at the lysosomal membrane
Source: Nat Commun. 2025 Nov 28;16:10731. doi: 10.1038/s41467-025-65760-6 (PMC12663577; doi:10.1038/s41467-025-65760-6)
Supplement: Supplementary file 1 — Supplementary information [file 41467_2025_65760_MOESM1_ESM.pdf]

Supplementary Fig. 1

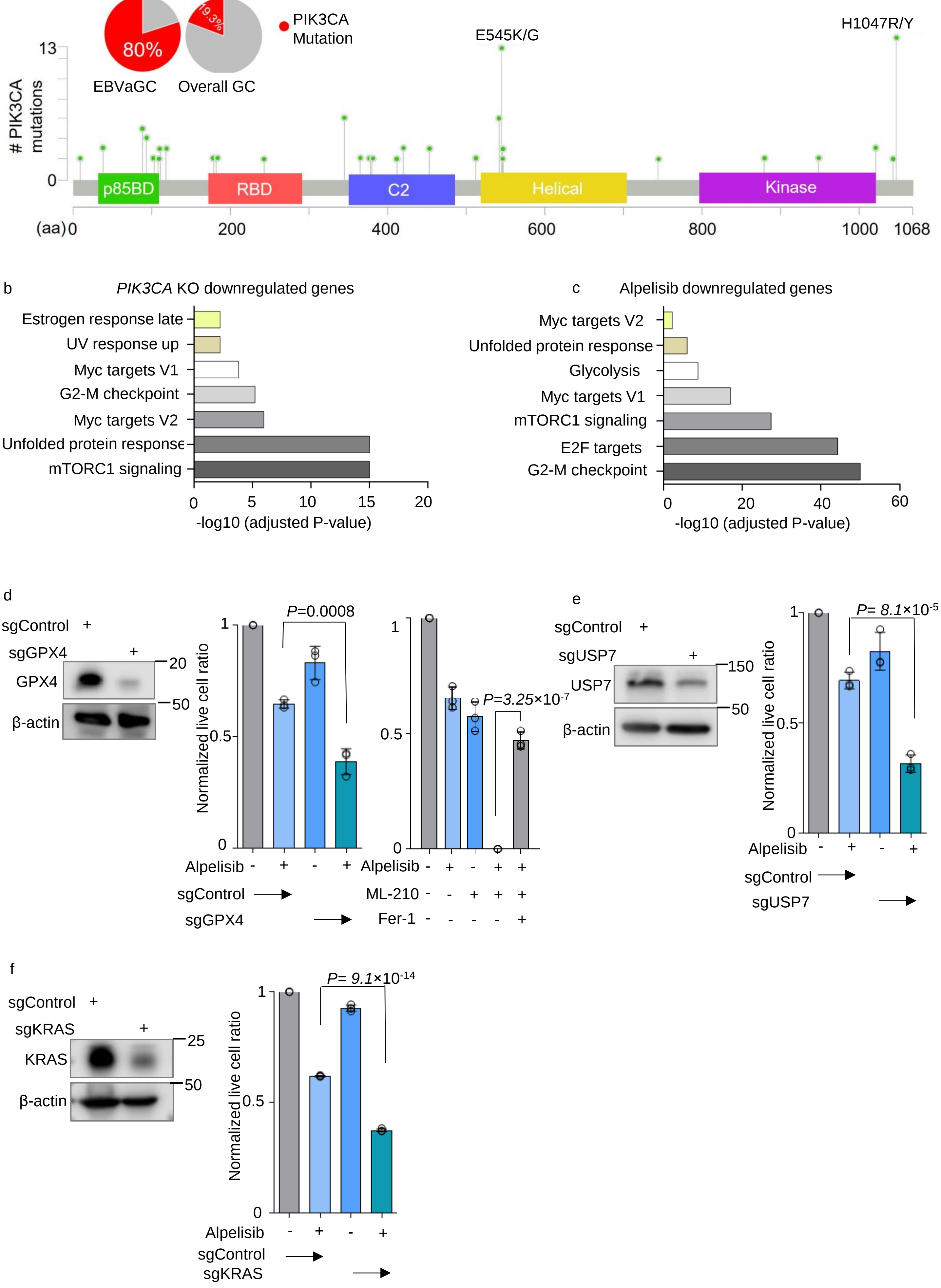

Supplementary Figure 1. Analysis of EBVaGC PI3K pathway target genes and alpelisib CRISPR screen hit validation.

(A) cBioPortal representation of gastric adenocarcinoma primary tumor tissue PIK3CA mutations sequenced by The Cancer Genome Atlas2. Shown are the # of missense mutations from 295 human tumors.

(B) MSigDB hallmark pathway analysis of genes that were downregulated in Cas9+ YCCEL1 expressing PIK3CA vs control sgRNAs. Differentially expressed genes (log_2 foldchange mRNA abundance < -0.5 and adjusted P-value< 0.05 between PIK3CA depleted versus control cells) were analyzed in Enrichr[97].

(C) MSigDB hallmark pathway analysis of genes downregulated in YCCEL1 treated with 5µM alpelisib vs DMSO for 72 hours. Differentially expressed genes (log_2 foldchange mRNA abundance < -0.5 and adjusted P-value< 0.05 between alpelisib vs DMSO treated cells) were analyzed in Enrichr[97].

(D) Validation of screen hit GPX4. Left, immunoblot analysis of WCL from YCCEL1 expressing control vs. GXP4 sgRNA. Middle, normalized live cell ratio mean ± SD values of YCCEL1 expressing the indicated sgRNA and treated with 0.5 µM alpelisib for 7 days. Right, normalized c cell ratio from *n*=3 independent replicates of YCCEL1 treated with 0.5 µM alpelisib, the GPX4 antagonist ML-210 (5µM) and Fer-1 (10µM) for 7-days. P-values were calculated by two-tailed one-way ANOVA.

(E) Validation of screen hit USP7. Left, immunoblot analysis of WCL from YCCEL1 expressing control vs. USP7 sgRNA. Right, normalized live cell ratio mean ± SD from *n*=3 independent replicates of YCCEL1 expressing the indicated control or USP7 sgRNA and treated with 0.5µM alpelisib for 7 days. P-values were calculated by two-tailed one-way ANOVA.

(F) Validation of screen hit KRAS. Left, immunoblot analysis of WCL from YCCEL1 expressing control vs. KRAS sgRNA for. Right, Mean normalized live cell ratios of YCCEL1 expressing the indicated sgRNA and treated with 0.5µM alpelisib for 7 days. Right, normalized live cell ratio mean ± SD values from *n*=3 independent replicates of YCCEL1 expressing the indicated sgRNA and treated with 0.5µM alpelisib for 7 days. P-values were calculated by two-tailed one-way ANOVA.

Blots are representative of *n*=3 independent experiments. Source data are provided as a Source Data file for (D)-(F).

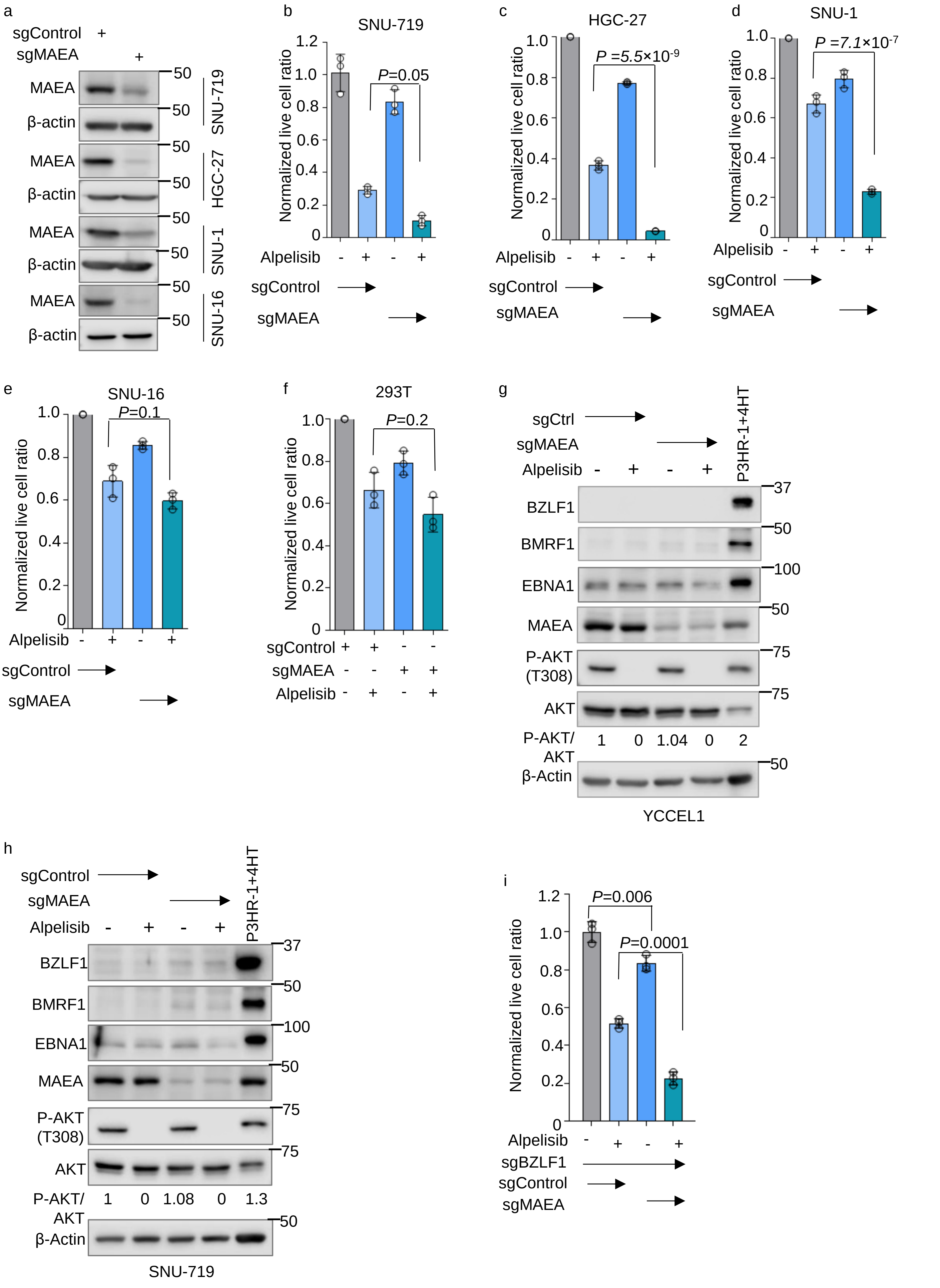

a
sgControl +
sgMAEA +
MAEA
β-actin
SNU-719
HGC-27
SNU-1
SNU-16
50
b
SNU-719
Normalized live cell ratio
P=0.05
Alpelisib - + - +
sgControl
sgMAEA
c
HGC-27
P =5.5×10-9
d
SNU-1
P =7.1×10-7
e
SNU-16
P=0.1
f
293T
P=0.2
sgControl + + - -
sgMAEA - - + +
Alpelisib - + - +
g
sgCtrl
sgMAEA
Alpelisib - + - +
P3HR-1+4HT
BZLF1
BMRF1
EBNA1
MAEA
P-AKT (T308)
AKT
P-AKT/ AKT 1 0 1.04 0 2
β-Actin
37
50
100
75
YCCEL1
h
sgControl
sgMAEA
Alpelisib - + - +
P3HR-1+4HT
P-AKT/ AKT 1 0 1.08 0 1.3
SNU-719
i
P=0.006
P=0.0001
Alpelisib - + - +
sgBZLF1
sgControl
sgMAEA

Supplementary Figure 2. Characterization of gastric carcinoma MAEA depletion and alpelisib synthetic effects.

(A) Immunoblot analysis of WCL from EBV-positive SNU-719, EBV-negative HGC-27, SNU-1 and SNU-16 gastric carcinoma expressing control or MAEA sgRNA. Blots are representative of *n*=3 independent experiments.

(B)-(F) Normalized mean ± SD live cell ratios from *n*=3 individual replicates of EBV-positive (B) SNU-719, (C) EBV-negative HGC-27, (D) EBV-negative SNU-1, (E) EBV-negative SNU-16, and (F) EBV-negative HEK-293T, that expressed control or MAEA sgRNAs and that were treated with alpelisib for 7 days, as indicated. Ns, non-significant. P-values were calculated by two-tailed one-way ANOVA. Source data are provided as a Source Data file.

(G) Immunoblot analysis of WCL from Cas9+ YCCEL1 expressing control or MAEA sgRNA and that were treated with 0.5µM alpelisib for 8 hours, as indicated. As a positive control, WCL were run in the last lane from the EBV+ Burkitt lymphoma cell line P3HR-1 with conditional EBV ZTA and RTA immediate early alleles that were activated by treatment with 4-hydroxytamoxifen for 24 hours to induce lytic reactivation. Source data are provided as a Source Data file.

(H) Immunoblot analysis of WCL from Cas9+ SNU-719 expressing control or MAEA sgRNA that were treated with 0.5µM alpelisib for 8 hours. WCL from 4HT-treated P3HR-1 with conditional immediate early ZTA and RTA alleles were included as a positive control as in (G). Source data are provided as a Source Data file.

(I) Normalized mean ± SD live cell ratios from *n*=3 replicates of Cas9+ YCCEL1 that expressed BZLF1 or MAEA sgRNAs and that were treated with 0.5µM alpelisib for 7 days, as indicated. P-values were calculated by two-tailed one-way ANOVA. Source data are provided as a Source Data file.

Blots are representative of *n*=3 independent experiments. Source data are provided as a Source Data file for (A)-(I).

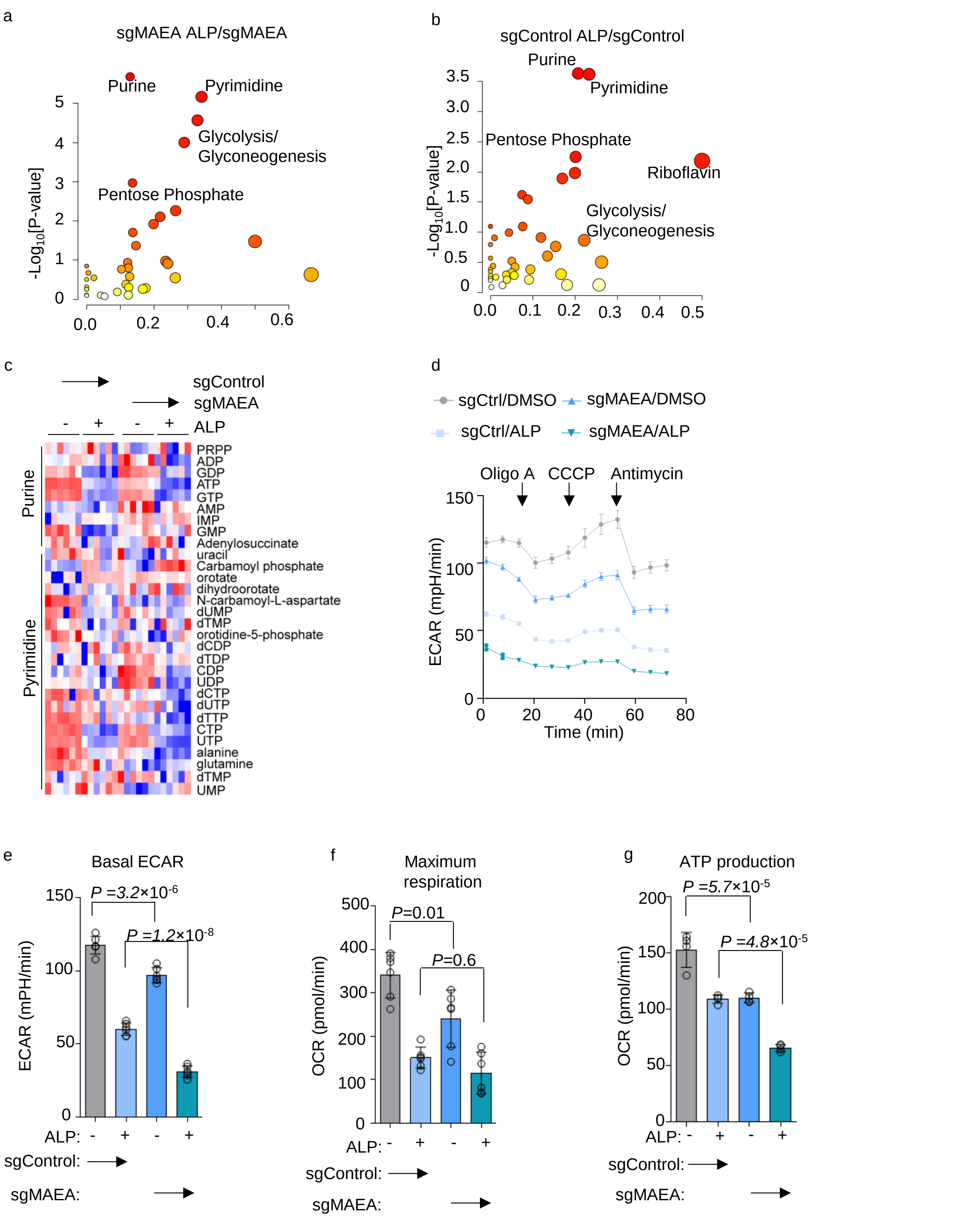
a
sgMAEA ALP/sgMAEA
Purine
Pyrimidine
Glycolysis/
Glyconeogenesis
Pentose Phosphate
-Log10[P-value]
b
sgControl ALP/sgControl
Purine
Pyrimidine
Pentose Phosphate
Riboflavin
Glycolysis/
Glyconeogenesis
-Log10[P-value]
c
sgControl
sgMAEA
ALP
Purine
Pyrimidine
PRPP
ADP
GDP
ATP
GTP
AMP
IMP
GMP
Adenylosuccinate
uracil
Carbamoyl phosphate
orotate
dihydroorotate
N-carbamoyl-L-aspartate
dUMP
dTMP
orotidine-5-phosphate
dCDP
dTDP
CDP
UDP
dCTP
dUTP
dTTP
CTP
UTP
alanine
glutamine
dTMP
UMP
d
sgCtrl/DMSO
sgMAEA/DMSO
sgCtrl/ALP
sgMAEA/ALP
Oligo A
CCCP
Antimycin
ECAR (mpH/min)
Time (min)
e
Basal ECAR
P =3.2×10-6
P =1.2×10-8
ECAR (mPH/min)
ALP:
sgControl:
sgMAEA:
f
Maximum respiration
P=0.01
P=0.6
OCR (pmol/min)
ALP:
sgControl:
sgMAEA:
g
ATP production
P =5.7×10-5
P =4.8×10-5
OCR (pmol/min)
ALP:
sgControl:
sgMAEA:

Supplementary Figure 3. Characterization of MAEA depletion and alpelisib effects on gastric cancer metabolism pathways.

(A) Metabolic pathway impact analysis of YCCEL1 MAEA depletion together with alpelisib as compared with MAEA depletion alone. Shown is the metabolic pathway impact map from LC/MS analyses (*n*=6 independent replicates) of YCCEL1 expressing MAEA and treated with alpelisib 0.5µM vs DMSO for 30 hours.

(B) Metabolic pathway impact analysis of alpelisib treatment on YCCEL1. Shown is the metabolic pathway impact map from LC/MS analyses (*n*=6 independent replicates) of YCCEL1 expressing control sgRNA and treated with alpelisib 0.5µM vs DMSO for 30 hours.

(C) Heatmap analyses of purine and pyrimidine pathway metabolite row Z-scores from LC/MS analysis of YCCEL1 cells expressing MAEA versus control sgRNAs and grown in the presence of 0.5µM alpelisib, as indicated. Z-scores show standard deviation(s) of metabolite abundances from the mean value in each row.

(D) Seahorse analysis of extracellular acidification rates (ECAR) of YCCEL1 that expressed control or MAEA sgRNA for 7 days, analyzed where indicated following addition of the ATP synthase in inhibitor oligomycin A, uncoupling agent CCCP or electron transport chain complex III inhibitor antimycin. Shown are the mean ± SEM from *n*=6 independent replicates.

(E) Mean ± SD basal ECAR rates from Seahorse analysis (n=6 independent replicates) presented in YCCEL1 as in Figure 2(I). P-values were calculated by two-tailed one-way ANOVA.

(F) Mean ± SD maximum respiration rates of YCCEL1 from Seahorse analysis (n=6 independent replicates) presented in Figure 2(I). P-values were calculated by two-tailed one-way ANOVA.

(G) Mean ± SD ATP production of YCCEL1 From Seahorse analysis (n=6 independent replicates) presented in Figure 2(I). P-values were calculated by two-tailed one-way ANOVA.

Source data are provided as a Source Data file for (D)-(G).

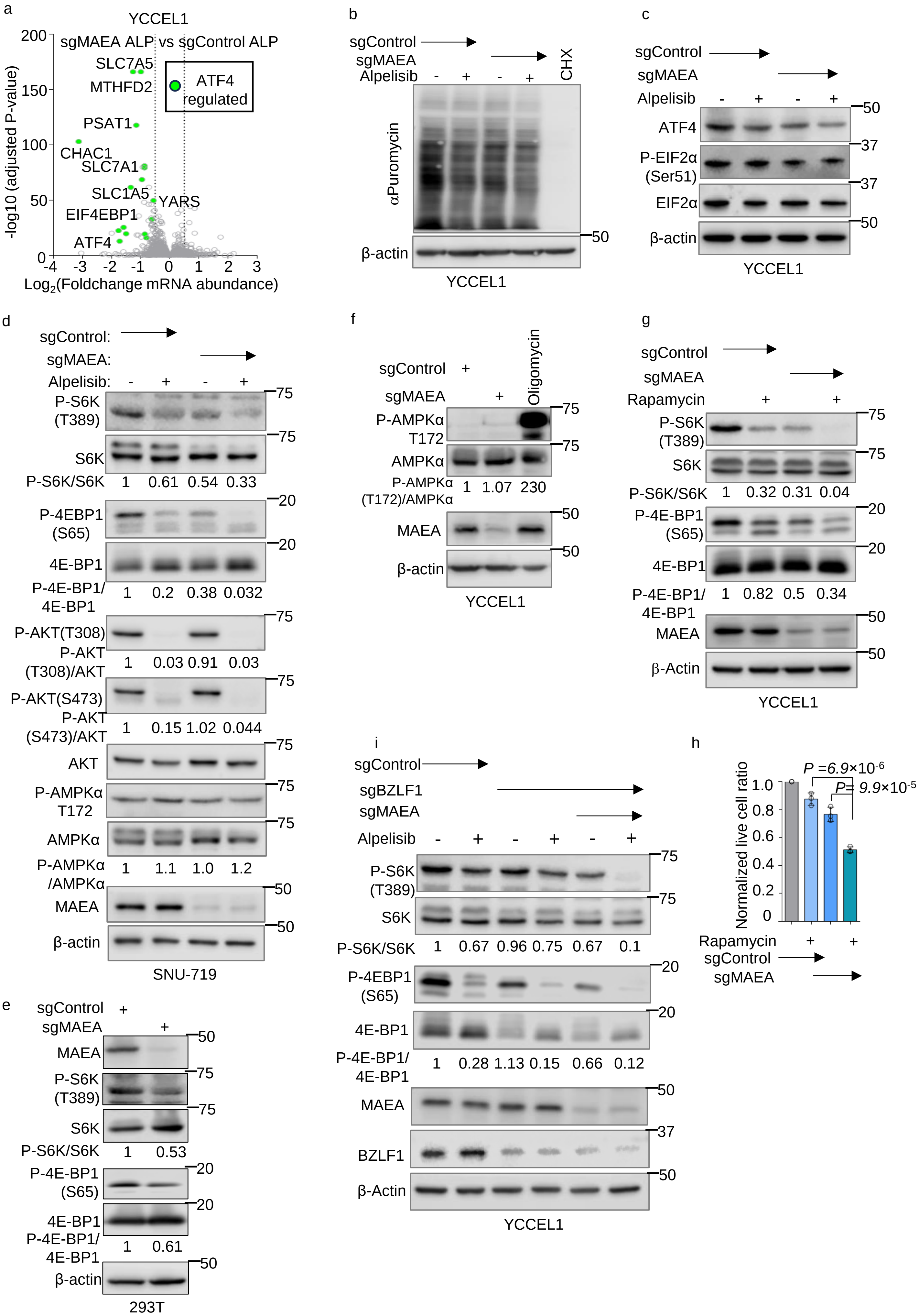
a
YCCEL1
sgMAEA ALP vs sgControl ALP
ATF4 regulated
SLC7A5
MTHFD2
PSAT1
CHAC1
SLC7A1
SLC1A5
YARS
EIF4EBP1
ATF4
-log10 (adjusted P-value)
Log2(Foldchange mRNA abundance)
b
sgControl
sgMAEA
Alpelisib - + - +
CHX
αPuromycin
β-actin
50
YCCEL1
c
sgControl
sgMAEA
Alpelisib - + - +
ATF4
P-EIF2α (Ser51)
EIF2α
β-actin
50
37
37
50
YCCEL1
d
sgControl:
sgMAEA:
Alpelisib: - + - +
P-S6K (T389)
S6K
P-S6K/S6K 1 0.61 0.54 0.33
P-4EBP1 (S65)
4E-BP1
P-4E-BP1/ 4E-BP1 1 0.2 0.38 0.032
P-AKT(T308)
P-AKT (T308)/AKT 1 0.03 0.91 0.03
P-AKT(S473)
P-AKT (S473)/AKT 1 0.15 1.02 0.044
AKT
P-AMPKα T172
AMPKα
P-AMPKα /AMPKα 1 1.1 1.0 1.2
MAEA
β-actin
SNU-719
e
sgControl +
sgMAEA +
MAEA
P-S6K (T389)
S6K
P-S6K/S6K 1 0.53
P-4E-BP1 (S65)
4E-BP1
P-4E-BP1/ 4E-BP1 1 0.61
β-actin
293T
f
sgControl +
sgMAEA +
Oligomycin
P-AMPKα T172
AMPKα
P-AMPKα (T172)/AMPKα 1 1.07 230
MAEA
β-actin
YCCEL1
g
sgControl
sgMAEA
Rapamycin + +
P-S6K (T389)
S6K
P-S6K/S6K 1 0.32 0.31 0.04
P-4E-BP1 (S65)
4E-BP1
P-4E-BP1/ 4E-BP1 1 0.82 0.5 0.34
MAEA
β-Actin
YCCEL1
i
sgControl
sgBZLF1
sgMAEA
Alpelisib - + - + - +
P-S6K (T389)
S6K
P-S6K/S6K 1 0.67 0.96 0.75 0.67 0.1
P-4EBP1 (S65)
4E-BP1
P-4E-BP1/ 4E-BP1 1 0.28 1.13 0.15 0.66 0.12
MAEA
BZLF1
β-Actin
YCCEL1
h
P =6.9×10-6
P= 9.9×10-5
Normalized live cell ratio
Rapamycin + +
sgControl
sgMAEA

Supplementary Figure 4. MAEA depletion inhibits mTOR.

(A) RNA-seq analysis of differentially-expressed genes in alpelisib treated YCCEL1 depleted of MAEA versus alpelisib treated YCCEL1 control cells. Shown is volcano plot analysis of YCCEL1 that expressed either MAEA or control sgRNAs and that were treated with alpelisib 5µM for 6h. Significantly changed ATF4 target genes are highlighted with green circles. Values for other genes are shown as empty circles.

(B) Immunoblot analysis of puromycin incorporation into newly synthesized polypeptides in YCCEL1 cells expressing control or MAEA sgRNA and treated with 0.5µM alpelisib for 8 hours. Cells were treated with cycloheximide (CHX, 50µg/mL) as a positive control.

(C) Immunoblot analysis of WCL from YCCEL1 expressing control or MAEA sgRNAs and treated with alpelisib 0.5µM for 8 hours.

(D) Immunoblot analysis of WCL from SNU-719 expressing control or MAEA sgRNAs for 7 days and treated with alpelisib 0.5µM for 8 hours, as indicated.

(E) Immunoblot analysis of WCL from HEK-293T electroporated with Cas9 ribonucloprotein complexes containing control or MAEA sgRNAs.

(F) Immunoblot analysis of WCL from YCCEL1 expressing control or MAEA sgRNAs or treated with oligomycin (1 µM) for 1 hour as a positive control.

(G) Immunoblot analysis of WCL from YCCEL1 expressing control or MAEA sgRNAs and treated with 5nM rapamycin.

(H) Mean ± SD normalized live cell ratio from *n*=3 independent replicates of YCCEL1 expressing control or MAEA sgRNA and treated with DMSO vs rapamycin (5nM) for 7 days. Live cell ratios were normalized to values measured in DMSO-treated cells expressing control sgRNA. P-values were calculated by two-tailed one-way ANOVA

(I) Immunoblot analysis of WCL from YCCEL1 expressing control, BZLF1 and/or MAEA targeting sgRNAs for 7 days and then treated with alpelisib 0.5µM for 8 hours.

Blots are representative of *n*=3 independent experiments. 'Source data are provided as a Source Data file for (B)-(I).

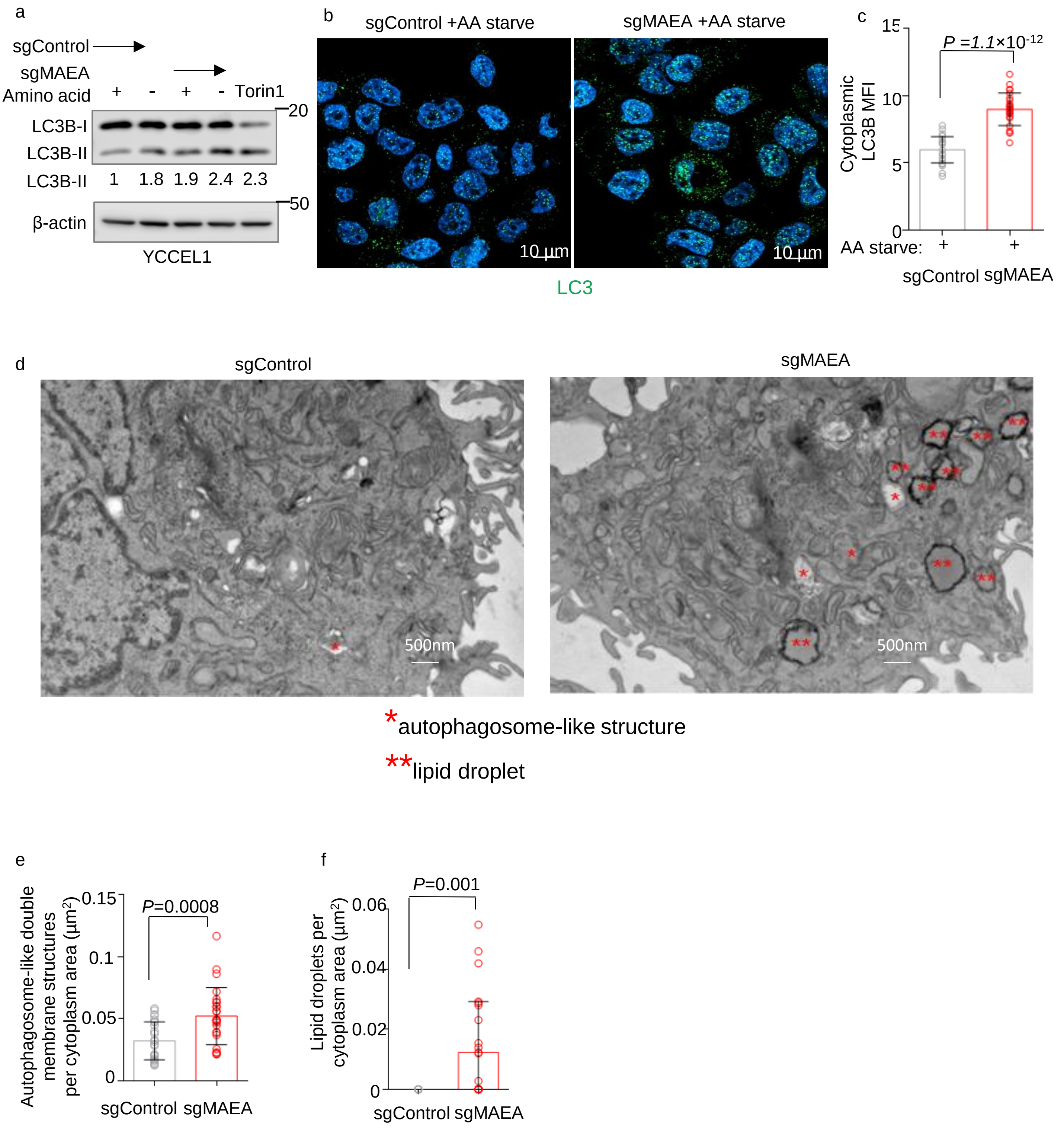
a
sgControl
sgMAEA
Amino acid + - + - Torin1
LC3B-I
LC3B-II
LC3B-II 1 1.8 1.9 2.4 2.3
20
50
β-actin
YCCEL1
b
sgControl +AA starve
sgMAEA +AA starve
10 μm
10 μm
LC3
c
Cytoplasmic LC3B MFI
15
10
5
0
P =1.1×10-12
AA starve: + +
sgControl sgMAEA
d
sgControl
sgMAEA
500nm
500nm
* autophagosome-like structure
** lipid droplet
e
Autophagosome-like double membrane structures per cytoplasm area (μm2)
0.15
0.1
0.05
0
P=0.0008
sgControl sgMAEA
f
Lipid droplets per cytoplasm area (μm2)
0.06
0.04
0.02
0
P=0.001
sgControl sgMAEA

Supplementary Figure 5. MAEA depletion increases autophagy and lipid droplet abundance in gastric carcinoma cells.

(A) Immunoblot analysis of WCL from YCCEL1 expressing control or MAEA sgRNAs and cultured in the absence or presence of amino acids for 50 minutes. As a positive control, lysates were included from cells treated with the mTOR inhibitor Torin 1 (100nM) for 3 hours in the last lane. Ratios of LC3B-II to b-actin are shown.

(B) Analysis of MAEA depletion effects on autophagy induction. Representative confocal microscopy images of YCCEL1 expressing control or MAEA sgRNAs that were cultured in the absence or presence of amino acids for 50 minutes. LC3 was stained with an Alexa Fluor 488 (green) conjugated antibody. Images are representative of n=3 replicates.

(C) Quantitation of LC3 abundance in control versus MAEA depleted and amino acid starved cells, as in (I). Shown are LC3 levels in *n*=25 randomly selected YCCEL1 cells, as in (I). Mean ± standard deviation of LC3 intensity are shown, with P-values calculated by by two-tailed Student's t-test.

(D) Transmission electron micrographs of YCCEL1 expressing control vs MAEA sgRNAs for 7 days. Representative images from >10 fields from *n*=3 replicates are shown. * indicate double membrane structures. ** indicate lipid droplets.

(E) Quantitation of autophagosome-like, double membrane structures per mm^2 of cytoplasm from transmission electron microscopy images of *n*=25 randomly selected cells, using methodology from[62, 99] with P-values calculated by two-tailed Student's t-test.

(F) Quantitation of lipid droplets per mm^2 of cytoplasm from electron microscopy images of 25 randomly selected cells from *n*=25 electron micrograph images with P-values calculated by by two-tailed Student's t-test.

Blots are representative of *n*=3 independent experiments. Source data are provided as a Source Data file for (A), (C), (E) and (F).

Supplementary Fig. 6

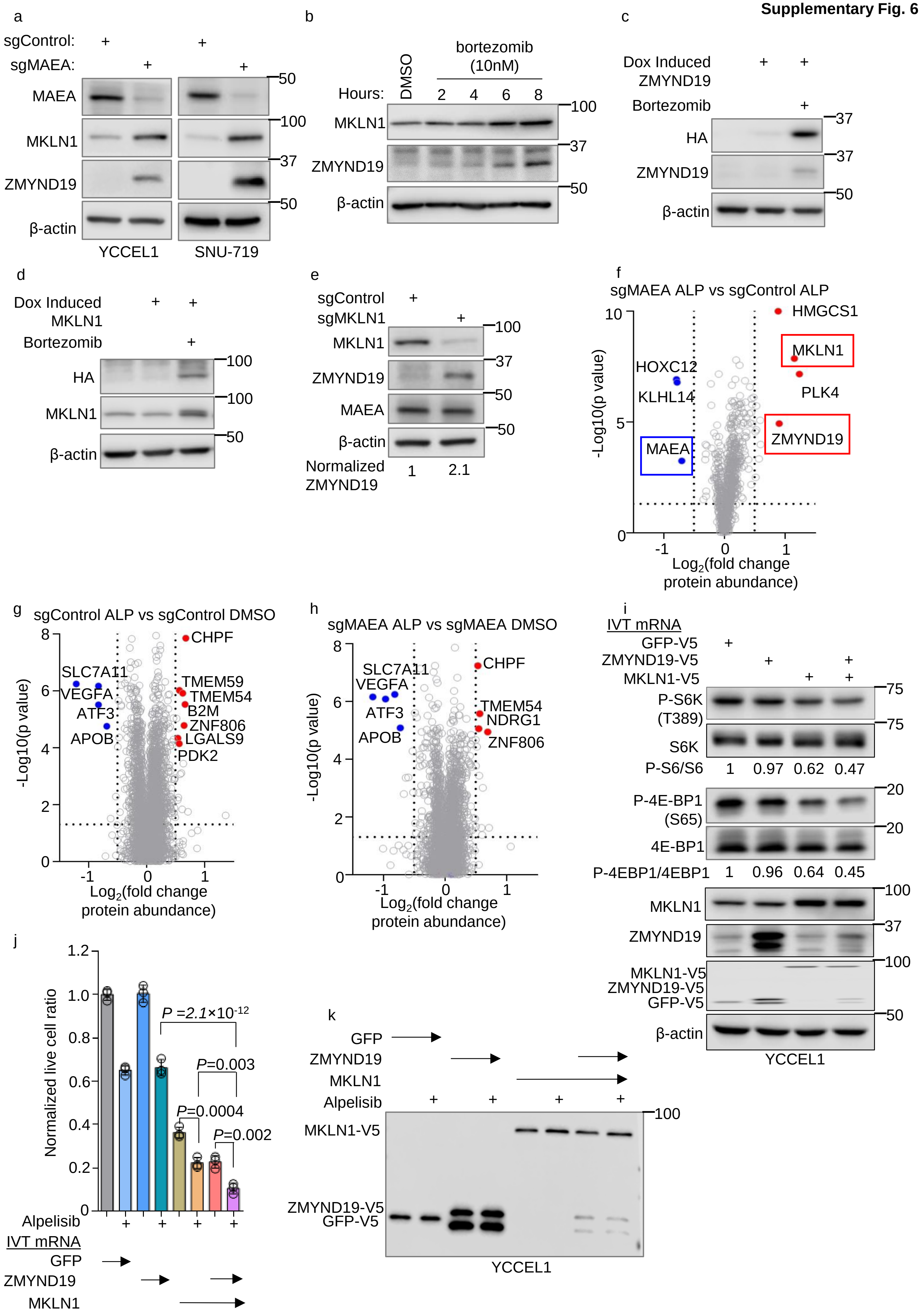

Supplementary Figure 6. CTLH substrates ZMYND19 and MKLN1 inhibit mTOR.

(A) Immunoblot analysis of WCL from YCCEL1 or SNU-719 expressing control or MAEA sgRNAs.

(B) Immunoblot analysis of WCL from YCCEL1 treated with DMSO or with bortezomib (10 nM) for the indicated time.

(C) Immunoblot analysis of WCL from YCCEL1 mock induced or induced for conditional HA-ZMYND19 cDNA expression by doxycycline (250 ng/mL) for 24 hours, in the absence or presence of bortezomib (10nM).

(D) Immunoblot analysis of WCL from YCCEL1 mock induced or induced for conditional HA-MKLN1 cDNA expression by doxycycline (250 ng/mL) for 24 hours, in the absence or presence of bortezomib (10nM).

(E) Immunoblot analysis of WCL from YCCEL1 that expressed control or MKLN1 sgRNAs.

(F) Volcano plot of whole cell proteomic analysis of MAEA depleted vs control cells to identify candidate YCCEL1 substrates in the presence of alpelisib. Shown are -log10 (P-value) y-axis vs Log2 (fold change in protein abundance) x-axis between YCCEL1 that expressed either MAEA or control sgRNA and treated with 0.5 µM alpelisib for 24 hours, from *n*=3 independent replicates.

(G) Volcano plot analysis as in (E) of YCCEL1 expressing control sgRNA and treated with alpelisib versus DMSO for 24 hours, from *n*=3 independent replicates.

(H) Volcano plot analysis as in (E) of YCCEL1 expressing MAEA sgRNA and treated with alpelisib versus DMSO for 24 hours, from *n*=3 independent replicates.

(I) Immunoblot analysis of WCL from YCCEL1 electroporated with *in vitro* transcribed (IVT) mRNAs encoding GFP, ZMYND19 or MKLN1. Cells were collected 3 hours post-electroporation. β-Actin was used as the loading control.

(J) Normalized live cell ratios of YCCEL1 (*n*=3 independent replicates) that were electroporated with GFP control, ZMYND19 and/or MKLN1 cDNAs and that were treated with alpelisib 0.5µM for 24 hours as in (I). P-values were calculated by two tailed one-way ANOVA.

(K) Immunoblot analysis of WCL from YCCEL1 that were electroporated with *in vitro* transcribed mRNAs encoding V5-epitope tagged control GFP, ZMYND19 or MKLN1 cDNAs.

Blots are representative of *n*=3 independent experiments. Source data are provided as a Source Data file for (A)-(E) and (I)-(K).

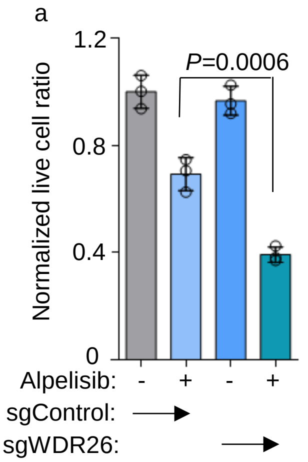
a
Normalized live cell ratio
1.2
0.8
0.4
0
P=0.0006
Alpelisib: - + - +
sgControl:
sgWDR26:

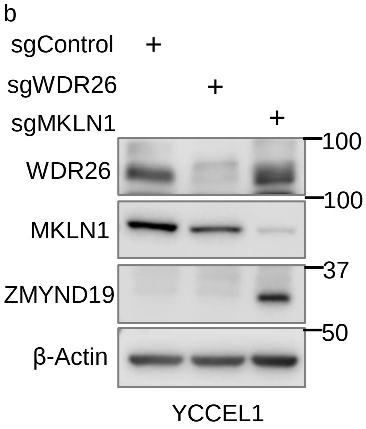
b
sgControl +
sgWDR26 +
sgMKLN1 +
WDR26
MKLN1
ZMYND19
β-Actin
100
100
37
50
YCCEL1

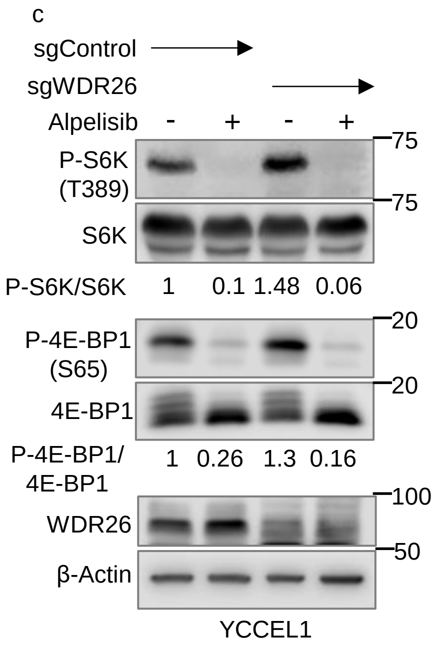
c
sgControl
sgWDR26
Alpelisib - + - +
P-S6K (T389)
S6K
P-S6K/S6K 1 0.1 1.48 0.06
P-4E-BP1 (S65)
4E-BP1
P-4E-BP1/ 4E-BP1 1 0.26 1.3 0.16
WDR26
β-Actin
75
75
20
20
100
50
YCCEL1

Supplementary Figure 7. WDR26 KO is synthetic lethal with alpelisib but does not stabilize MKLN1, ZMYND19 or impair mTOR activity.

(A) Normalized mean ± SD live cell ratios from *n*=3 replicates of YCCEL1 that expressed control or WDR26 targeting sgRNAs and that were treated with 0.5µM alpelisib for 7 days, as indicated. P-values were calculated by two-tailed one-way ANOVA.

(B) Immunoblot analysis of WCL from YCCEL1 that expressed control, WDR26 or MKLN1 targeting sgRNA.

(C) Immunoblot analysis of WCL from Cas9+ YCCEL1 expressing control vs. WDR26 sgRNA for 7 days and treated with alpelisib 0.5µM for 8 hours, as indicated. Source data are provided as a Source Data file.

Blots are representative of *n*=3 independent experiments. Source data are provided as a Source Data file for (A)-(C).

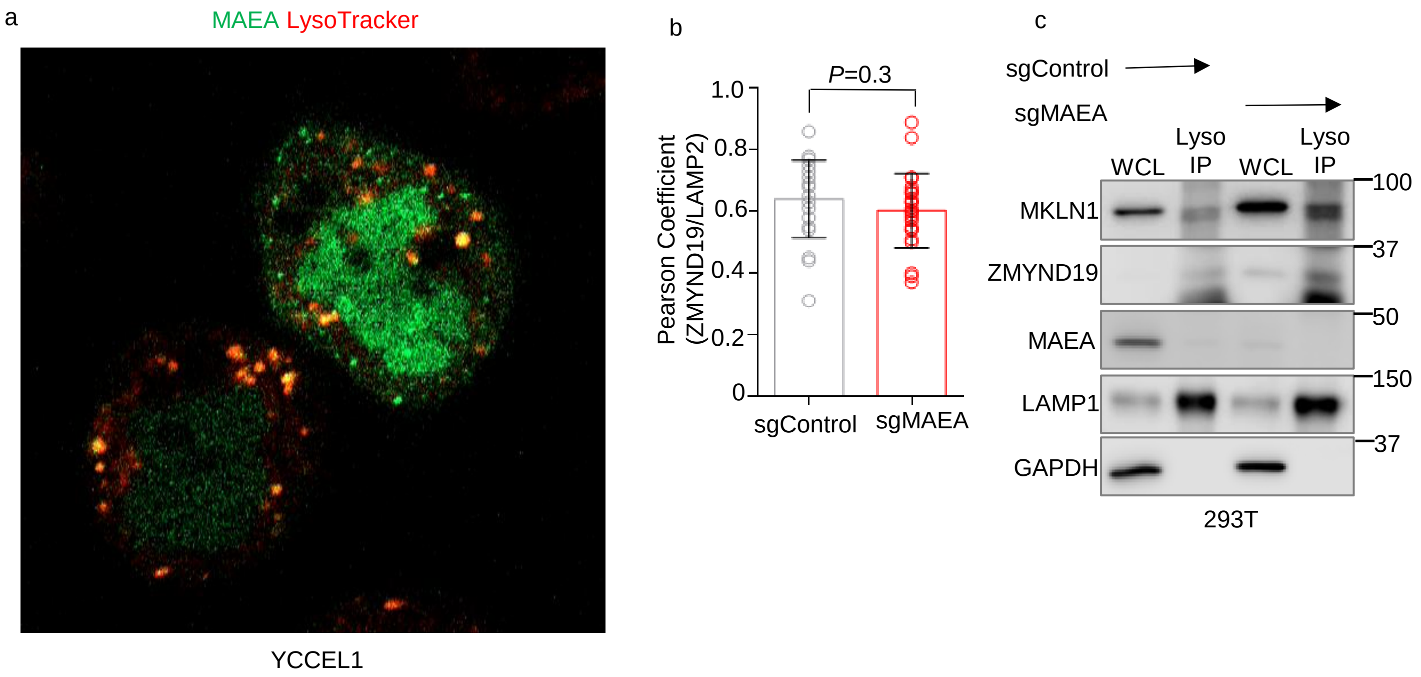
a
MAEA LysoTracker
YCCEL1
b
P=0.3
1.0
0.8
0.6
0.4
0.2
0
Pearson Coefficient (ZMYND19/LAMP2)
sgControl
sgMAEA
c
sgControl
sgMAEA
WCL
Lyso IP
WCL
Lyso IP
MKLN1
ZMYND19
MAEA
LAMP1
GAPDH
100
37
50
150
37
293T

Supplementary Figure 8. CTLH substrates ZMYND19 and MKLN1 associate with lysosomes upon CTLH inhibition.

(A) Live cell confocal microscopy imaging of the subcellular distribution of GFP-tagged MAEA and LysoTracker red stained lysosomes, as in Fig. 4A.

(B) Analysis of ZMYND19 and LAMP2 co-localization, using *n*=25 cells as in Fig. 4f. P-values were calculated by two-tailed Student's t-test.

(C) Immunoblot analysis of WCL or LysoIP from Cas9+ 293T expressing control or MAEA sgRNAs.

Blots are representative of *n*=3 independent experiments. Source data are provided as a Source Data file for (B) and (C).

Supplementary Fig. 9

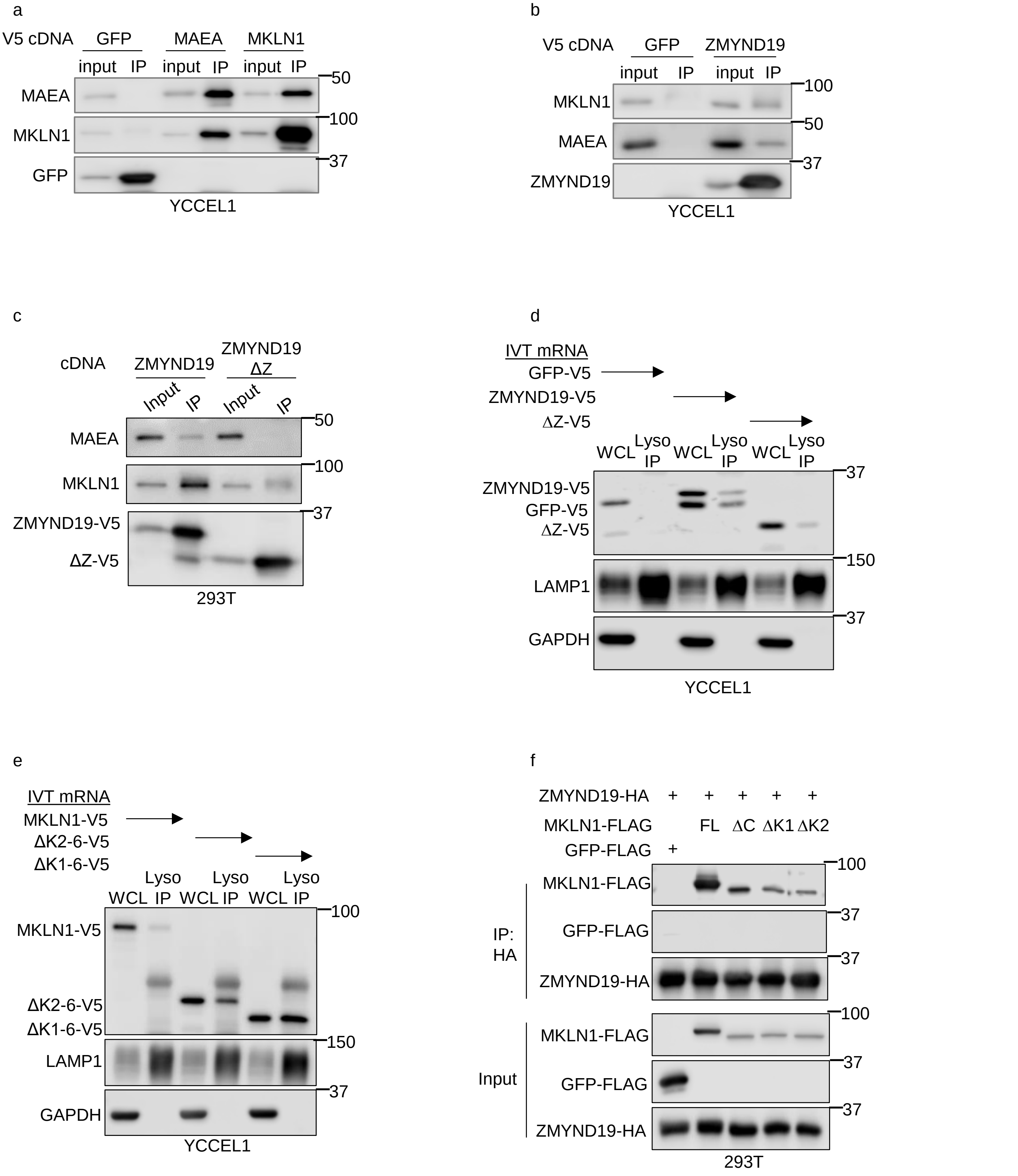

Supplementary Figure 9. ZMYND19/MKLN1 associate and block mTOR.

(A) MAEA and MKLN1 co-immunoprecipitation analysis. Immunoblots of 5% input vs anti-V5 immunopurified V5-GFP, V5-MAEA or V5-MKLN1 from YCCEL1 that stably expressed the indicated cDNAs.

(B) MAEA and ZMYND19 co-immunoprecipitation analysis. Immunoblots of 5% input vs anti-V5 immunopurified V5-GFP or V5-ZMYND9 from YCCEL1 that stably expressed the indicated cDNAs.

(C) Analysis of ZMYND19 zinc finger role in association with MKLN1 and MAEA. Immunoblots of 5% input vs anti-V5 immunopurified full length versus ΔZ ZMYND19 from 293T that transiently expressed the indicated cDNAs.

(D) Analysis of ZMYND19 zinc finger lysosome association roles. Immunoblot analysis of WCL and LysoIP samples from YCCEL1 expressing TMEM192-HA and electroporated with the indicated in vitro transcribed (IVT) mRNAs encoding V5-tagged GFP, full length (FL) or zinc finger domain deleted (ΔZ) ZMYND19. WCL and LysoIP samples were prepared 3 hours after electroporation.

(E) Analysis of MKLN1 Kelch (K) domain roles in lysosome association. Immunoblot analysis of WCL vs LysoIP samples from YCCEL1 electroporated with the indicated IVT mRNAs encoding full length vs C-terminal MKLN1 deletion mutants lacking Kelch domains 2-6 (ΔK2-6) or 1-6 (ΔK1-6). WCL and LysoIP samples were prepared three hours after electroporation.

(F) Analysis of MKLN1 domains important for association with ZMYND19. Immunoblot analysis of 5% input vs anti-FLAG immunopurified complexes from 293T that transiently expressed the indicated FLAG-tagged GFP, or MKLN1 constructs together with HA-tagged ZMYND19 for 24 hours. Flag-GFP was used as negative control.

Blots are representative of *n*=3 independent experiments. Source data are provided as a Source Data file for (A)-(F).

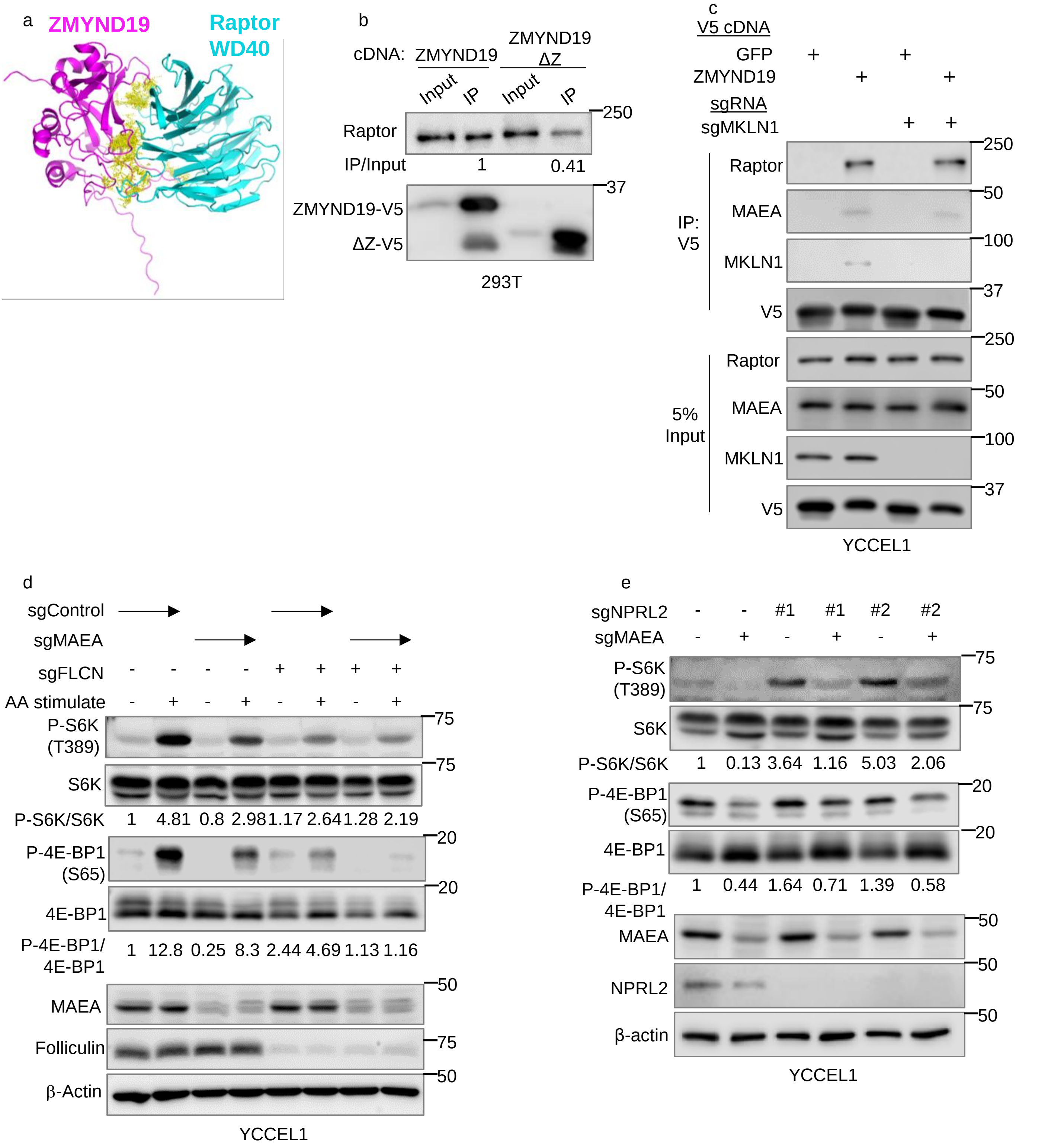
a
ZMYND19
Raptor
WD40
b
cDNA:
ZMYND19
ZMYND19
ΔZ
Input
IP
Input
IP
Raptor
IP/Input
1
0.41
ZMYND19-V5
ΔZ-V5
250
37
293T
c
V5 cDNA
GFP
ZMYND19
sgRNA
sgMKLN1
IP:
V5
Raptor
MAEA
MKLN1
V5
5%
Input
Raptor
MAEA
MKLN1
V5
250
50
100
37
YCCEL1
d
sgControl
sgMAEA
sgFLCN
AA stimulate
P-S6K
(T389)
S6K
P-S6K/S6K
1 4.81 0.8 2.98 1.17 2.64 1.28 2.19
P-4E-BP1
(S65)
4E-BP1
P-4E-BP1/
4E-BP1
1 12.8 0.25 8.3 2.44 4.69 1.13 1.16
MAEA
Folliculin
β-Actin
75
20
50
YCCEL1
e
sgNPRL2
- - #1 #1 #2 #2
sgMAEA
- + - + - +
P-S6K
(T389)
S6K
P-S6K/S6K
1 0.13 3.64 1.16 5.03 2.06
P-4E-BP1
(S65)
4E-BP1
P-4E-BP1/
4E-BP1
1 0.44 1.64 0.71 1.39 0.58
MAEA
NPRL2
β-actin
75
20
50
YCCEL1

Supplementary Figure 10. Association of ZMYND19, MKLN1 and Raptor.

(A) Alphafold multimer 2 model of ZMYND19 and Raptor association at 3.5 Å. Raptor is colored teal and the ZMYD19 is colored magenta. A predicted interaction surface is detailed in yellow.

(B) Analysis of whether the ZMYND19 zinc finger is necessary for association with Raptor. Immunoblots of 5% input vs anti-V5 immunopurified full length versus ΔZ ZMYND19 from 293T that transiently expressed the indicated cDNAs. Shown also are ratios of Raptor in immunopurified versus WCL samples, with values in wildtype ZMYND19 expressing cells set to 1.

(C) Analysis of MKLN1 roles in association between ZMYND19 and Raptor. Immunoblots of 5% input versus ant-V5 immuno-purified complexes from YCCEL1 cells that transiently expressed GFP or ZMYND19 cDNAs and that also expressed control or MKLN1 sgRNAs.

(D) Analysis of whether folliculin is necessary for mTORC1 inhibition upon MAEA depletion. Immunoblot analysis of WCL from YCCEL1 expressing the indicated control, MAEA and/or folliculin (FLCN) sgRNAs and that had been amino acid starved for 50 minutes and then cultured for 10 minutes in medium without or with amino acids.

(E) Analysis of whether the GATOR1 catalytic subunit NPRL2 is necessary for mTORC1 inhibition upon MAEA depletion. Immunoblot analysis of WCL from YCCEL1 expressing the indicted MAEA or independent NPRL2 (#1 or #2) sgRNAs.

Blots are representative of *n*=3 independent experiments. Source data are provided as a Source Data file for (B)-(E).

Supplementary Fig. 11

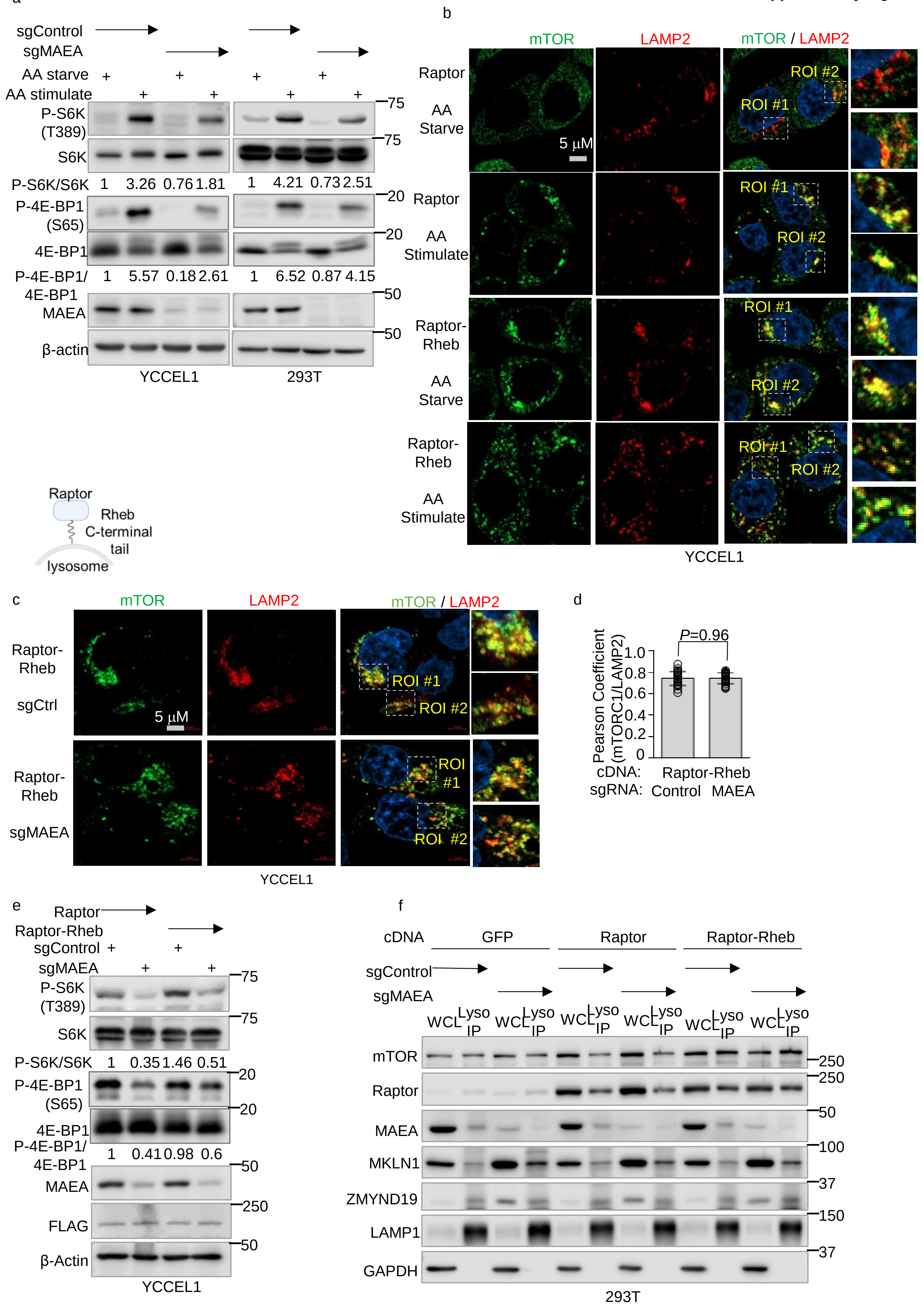

Supplementary Figure 11. ZMYND19 and MKLN1 don't block mTORC1 recruitment to lysosomal membrane.

(A) Analysis of MAEA depletion effects on amino acid stimulated mTORC1 activity. Immunoblot analysis of WCL from Cas9+ YCCEL1 (left) or 293T (right) that expressed control or MAEA sgRNAs and that were amino acid (AA) starved for 50 minutes and then cultured in medium in the absence or presence of amino acids for 10 minutes, as indicated.

(B) Analysis of Raptor-Rheb effects on mTORC1 subcellular localization in amino acid starved versus replete conditions. Confocal microscopy analysis of YCCEL1 that expressed Raptor or Raptor-Rheb cDNAs and that were amino acid (AA) starved for 50 minutes and then cultured in medium in the absence or presence of amino acids for 10 minutes, as indicated. Zoomed images of representative cells are shown below.

(C) Analysis of whether constitutively lysosomal membrane targeted Raptor can bypass mTORC1 blockade by MAEA depletion. Confocal microscopy of YCCEL1 expressing control or MAEA sgRNAs together with a Raptor fused to the Rheb C-terminal tail 15 amino acids, which serve as a lysosomal targeting sequence to constitutively drive Raptor to the lysosomes in an amino acid independent manner84 (as demonstrated in the model to the right). mTOR and lysosomal marker LAMP2 were stained with Alexa Fluor 488 (green) or Alexa Fluor 595 (red) conjugated antibodies, respectively. Zooms of two regions of interest (ROI) are shown to the right of each panel. Created in BioRender. Guo, R. (2025) https://BioRender.com/t1ugr37

(D) Analysis of mTORC1 and LAMP2 co-localization, using *n*=25 cells as in (C). P values were calculated by two-tailed Student's t-test.

(E) Analysis of Raptor-Rheb bypass of mTORC1 inhibition by MAEA depletion. Immunoblots of WCL from YCCEL1 that expressed Raptor or Raptor-Rheb cDNAs (as in C).

(F) Analysis of Raptor-Rheb bypass effects on mTORC1 lysosomal recruitment and phosphorylation in control vs MAEA depleted cells. Immunoblot analysis of WCL for LysoIP obtained from 293T that expressed cDNAs encoding GFP, Raptor or Raptor-Rheb together with control or MAEA sgRNAs.

Blots are representative of *n*=3 independent experiments. Source data are provided as a Source Data file for (A) and (D)-(F).

Supplementary Fig. 12

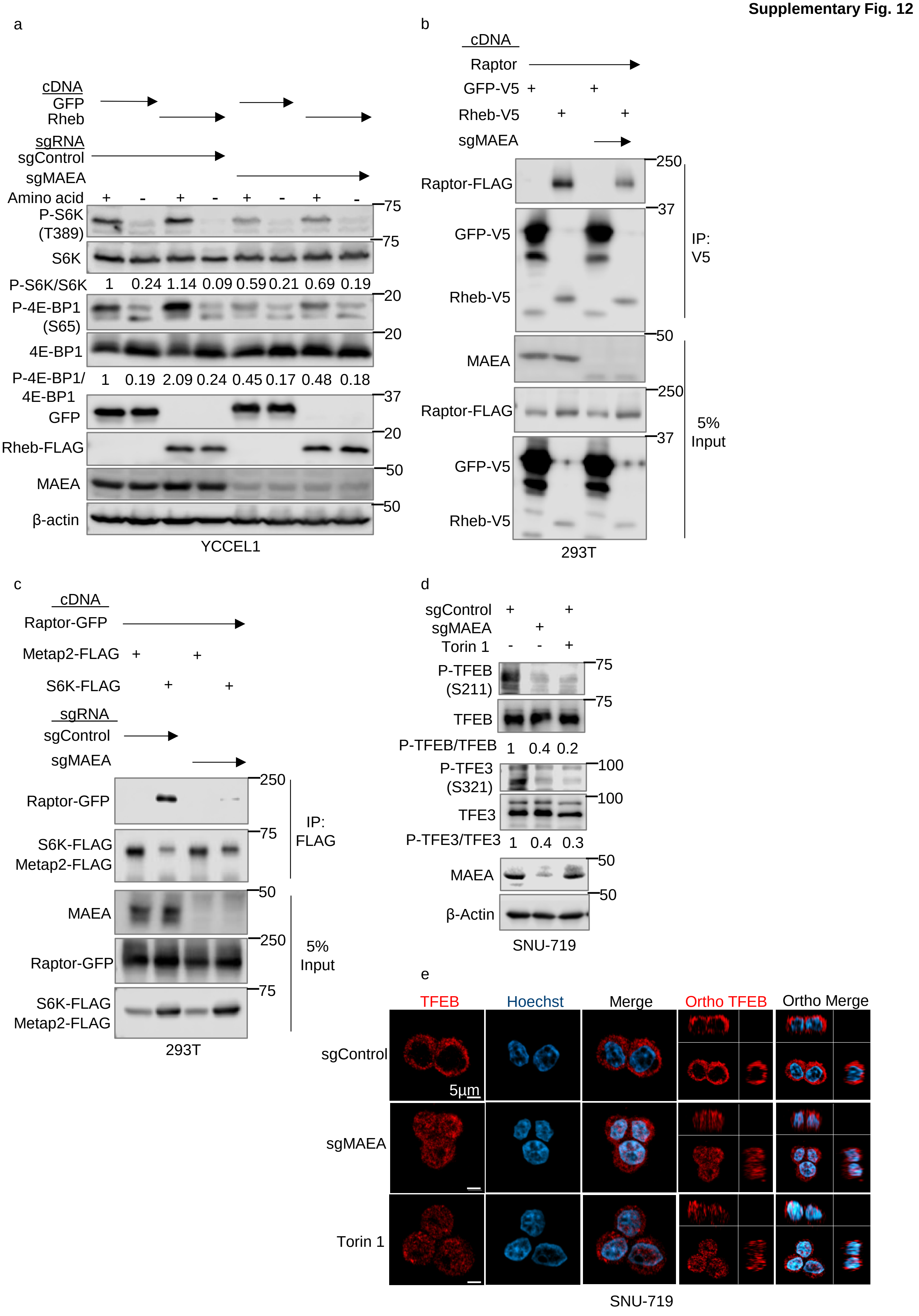

Supplementary Figure 12. ZMYND19/MKLN1 impair the mTORC1 interactions with Rheb and its downstream substrates

(A) Analysis of Rheb overexpression effects on MAEA knockout mediated mTOR inhibition. Immunoblot analysis of WCL from YCCEL1 expressing GFP or Rheb cDNA and either control or MAEA sgRNAs, cultured in medium in the absence or presence of amino acids for 50 minutes.

(B) Analysis of MAEA depletion effects on Raptor association with Rheb. Immunoblot analysis of 5% input versus anti-V5 immuno-purified complexes from 293T control or MAEA KO single cell clones that were transiently transfected with Raptor-FLAG, GFP-V5 or Rheb-V5 expression vectors, as indicated.

(C) Analysis of MAEA depletion effects on Raptor association with mTORC1 substrate S6K. Immunoblot analysis of 5% input versus anti-FLAG immuno-purified complexes from 293T control or MAEA KO single cell clones that were transiently transfected with Raptor-GFP, control Metap2-FLAG or S6K-FLAG expression vectors, as indicated.

(D) Immunoblot analysis of WCL from SNU-719 cells expressing control or MAEA sgRNAs for 7 days and treated with Torin 1 (100nM) for 3 hours, as indicated.

(E) Confocal microscopy images of TFEB, nuclear Hoechst or merged TFEB/Hoechst staining from SNU-719 cells treated as in (D). Ortho refers to image reconstruction using orthogonal views that integrated XY projections with XZ and YZ cross-sections to confirm three-dimensional localization.

Blots are representative of *n*=3 independent experiments. Source data are provided as a Source Data file for (A)-(D).
