# Supplementary material for: The CTLH ubiquitin ligase substrates ZMYND19 and MKLN1 negatively regulate mTORC1 at the lysosomal membrane
Source: Nat Commun. 2025 Nov 28;16:10731. doi: 10.1038/s41467-025-65760-6 (PMC12663577; doi:10.1038/s41467-025-65760-6)
Supplement: Supplementary file 3 — Description of Additional Supplementary Files [file 41467_2025_65760_MOESM3_ESM.docx]

Supplementary Data 1. RNAseq analysis of YCCEL1 treated with DMSO or alpelisib and expressing sgcontrol or sgPIK3CA

Supplementary Data 2. YCCEL1 genome-wide CRISPR-Cas9 screen. Cas9+ YCCEL1 transduced with the Brunello sgRNA library were selected with puromycin and cultured in DMSO or alpelisib.

Supplementary Data 3. Cell cycle analysis of propidium iodide stained YCCEL1 expressing MAEA vs control sgRNAs treated with DMSO or alpelisib.

Supplementary Data 4. Metabolic pathway impact analysis of YCCEL1 expressing MAEA vs control sgRNAs treated with DMSO or alpelisib.

Supplementary Data 5. 2-NBDG uptake in YCCEL1 expressing Control (Ctrl) or MAEA sgRNAs and treated with DMSO or alpelisib (ALP).

Supplementary Data 6. RNAseq analysis of YCCEL1 expressing MAEA vs control sgRNAs treated with alpelisib or DMSO.

Supplementary Data 7. YCCEL1 whole cell proteomic analysis of MAEA depleted vs control cells treated with alpelisib or DMSO.

Supplementary Data 8. Oligonucleotide information used in the study.

Supplementary Movie 1. Live cell image of YCCEL1 overexpressed MAEA-GFP stained with LysoTracker red.
