# Supplementary material for: The CTLH ubiquitin ligase substrates ZMYND19 and MKLN1 negatively regulate mTORC1 at the lysosomal membrane
Source: Nat Commun. 2025 Nov 28;16:10731. doi: 10.1038/s41467-025-65760-6 (PMC12663577; doi:10.1038/s41467-025-65760-6)
Supplement: Supplementary file 13 — Reporting Summary [file 41467_2025_65760_MOESM13_ESM.pdf]

nature portfolio

Corresponding author(s): Benjamin E. Gewurz

Last updated by author(s): Oct 8, 2025

Reporting Summary

Nature Portfolio wishes to improve the reproducibility of the work that we publish. This form provides structure for consistency and transparency in reporting. For further information on Nature Portfolio policies, see our Editorial Policies and the Editorial Policy Checklist.

Statistics

For all statistical analyses, confirm that the following items are present in the figure legend, table legend, main text, or Methods section.

n/a	Confirmed	
☐	☒	The exact sample size (*n*) for each experimental group/condition, given as a discrete number and unit of measurement
☐	☒	A statement on whether measurements were taken from distinct samples or whether the same sample was measured repeatedly
☐	☒	The statistical test(s) used AND whether they are one- or two-sided<br>*Only common tests should be described solely by name; describe more complex techniques in the Methods section.*
☒	☐	A description of all covariates tested
☐	☒	A description of any assumptions or corrections, such as tests of normality and adjustment for multiple comparisons
☐	☒	A full description of the statistical parameters including central tendency (e.g. means) or other basic estimates (e.g. regression coefficient) AND variation (e.g. standard deviation) or associated estimates of uncertainty (e.g. confidence intervals)
☐	☒	For null hypothesis testing, the test statistic (e.g. F, t, r) with confidence intervals, effect sizes, degrees of freedom and P value noted<br>*Give P values as exact values whenever suitable.*
☒	☐	For Bayesian analysis, information on the choice of priors and Markov chain Monte Carlo settings
☒	☐	For hierarchical and complex designs, identification of the appropriate level for tests and full reporting of outcomes
☐	☒	Estimates of effect sizes (e.g. Cohen's *d*, Pearson's *r*), indicating how they were calculated

Our web collection on statistics for biologists contains articles on many of the points above.

Software and code

Policy information about availability of computer code

Data collection	For flow cytometry acquisition: BD CellQuest Pro (v 6); For immunoblot acquisition: ImageStudioLite Odyssey software (v 5.2.5); For confocal microscopy and live cell imaging: Zeiss LSM 800. For mRNA electroporation: The Neon Transfection System. For Seahorse analysis: Agilent seahorse XF96 analyzer.
Data analysis	The STARS algorithm was applied to calculate hit statistical significance, using a stringent cutoff of q < 0.05 (p-value adjusted for the False Discovery Rate). All blots, FACS, and immunofluorescence images show representative images from n =3 replicates. All bar and line graphs are shown as mean ± standard deviation (SD) of n = 3 replicates. Statistical significance between indicated groups was determined using the one-way AVOVA or student’s t-test with GraphPad Prism 8 software, where NS denotes not significant (p > 0.05), * p < 0.05, ** p < 0.01, and *** p < 0.001.

For manuscripts utilizing custom algorithms or software that are central to the research but not yet described in published literature, software must be made available to editors and reviewers. We strongly encourage code deposition in a community repository (e.g. GitHub). See the Nature Portfolio guidelines for submitting code & software for further information.

Data

Policy information about availability of data

All manuscripts must include a data availability statement. This statement should provide the following information, where applicable:

- Accession codes, unique identifiers, or web links for publicly available datasets
- A description of any restrictions on data availability
- For clinical datasets or third party data, please ensure that the statement adheres to our policy

All data relevant to the findings of this study are provided within the article and its accompanying supplementary information files. RNAseq data has been deposited into the NIH GEO omnibus (GEO accession number: GSE271761 and GSE271762) and will be released upon publication.

Research involving human participants, their data, or biological material

Policy information about studies with human participants or human data. See also policy information about sex, gender (identity/presentation), and sexual orientation and race, ethnicity and racism.

Reporting on sex and gender	n/a
Reporting on race, ethnicity, or other socially relevant groupings	n/a
Population characteristics	n/a
Recruitment	n/a
Ethics oversight	n/a

Note that full information on the approval of the study protocol must also be provided in the manuscript.

Field-specific reporting

Please select the one below that is the best fit for your research. If you are not sure, read the appropriate sections before making your selection.

[x] Life sciences [ ] Behavioural & social sciences [ ] Ecological, evolutionary & environmental sciences

For a reference copy of the document with all sections, see nature.com/documents/nr-reporting-summary-flat.pdf

Life sciences study design

All studies must disclose on these points even when the disclosure is negative.

Sample size	For the CRISPR screen, 40 million cells were infected, ensuring an average of 500 cells were infected by each lentivirus in the pool (each sgRNA was represented by 500 cells in the input library, based on statistical calculations from PMID 26780180). For subsequent experiments, we generally used n=3 independent biological replicates.
Data exclusions	No data was excluded.
Replication	All data reflect biological replicates, as detailed in the figure legends and methods. Error bars indicate the standard deviation derived from a minimum of three biological replicates. Results were reproducible across different replicates.
Randomization	In all experiments, control samples were collected in parallel with those undergoing treatment/perturbation. Samples were randomly assigned to control or treatment groups and subjected to identical conditions, except for the specific treatment or perturbation being tested. Detailed protocols for each experiment are provided in the Methods.
Blinding	Due to the necessity of treating samples with specific lentiviruses and collecting them at defined time points, investigators were not blinded during sample collection. However, the experiments were consistently performed with biological replicates across multiple years.

Reporting for specific materials, systems and methods

We require information from authors about some types of materials, experimental systems and methods used in many studies. Here, indicate whether each material, system or method listed is relevant to your study. If you are not sure if a list item applies to your research, read the appropriate section before selecting a response.

Materials & experimental systems

n/a	Involved in the study
	[x] Antibodies
	[x] Eukaryotic cell lines
[x]	Palaeontology and archaeology
[x]	Animals and other organisms
[x]	Clinical data
[x]	Dual use research of concern
[x]	Plants

Methods

n/a	Involved in the study
[x]	ChIP-seq
	[x] Flow cytometry
[x]	MRI-based neuroimaging

Antibodies

Antibodies used

Antibodies against the following were purchased from Cell Signaling Technology: Phospho-Akt (Ser473) (D9E, Cat#4060S), Phospho-Akt (Thr308) (244F9, Cat#4056S), Akt (Cat#9272S), GPX4 (Cat#52455S), Phospho-p70 S6 Kinase (Thr389) (108D2, Cat#9234S), p70 S6 Kinase (Cat#9202S), Phospho-4E-BP1 (Ser65) (Cat#9451S), 4E-BP1 (53H11, Cat#9644S), V5-Tag (D3H8Q, Cat#13202S), Phospho-AMPKα (Thr172) (40H9, Cat#2535S), AMPKα (D5A2, Cat#2532S), Phospho-eIF2α (Ser51, Cat#9721S), eIF2α (D7D3, Cat#5324S), LC3B (D11, Cat#3868S), Raptor (24C12, Cat#2280S), HA-Tag (C29F4, Cat#3724S), GAPDH (D16H11, Cat#5174S), Golgin-97 (D8P2K, Cat#13192S), Calreticulin (D3E6, Cat#12238S), VDAC1 (D73D12, Cat#4661S), Myc-Tag (9B11, Cat#2276S), Myc-Tag (71D10, Cat#2278S), DYKDDDDK Tag (Cat#2368S), NPRL2 (D8K3X, Cat#37344S), FLCN (D14G9, Cat#3697S), mTOR (7C10, Cat#2983S), Phospho-TFEB (Ser211) (E9S8N, Cat37681S), TFEB (D2O7D, Cat#91767S), Phospho-TFE3 (Ser321) (23816S), TFE3 (F3X8T, Cat#19950T), anti-Rabbit IgG HRP-coupled secondary antibody (Cat#7074S), anti-Mouse IgG HRP-coupled secondary antibody (Cat#7076S), anti-Rat IgG HRP-coupled secondary antibody (Cat#7077S). Antibodies against the following were purchased from Proteintech: KRAS (Cat# 12063-1-AP) and USP7 (Cat# 66514-1-Ig). Antibodies against the following were purchased from Biolegend: Beta-actin (Cat#664802) and ATF4 (Cat#693901). Anti-MAEA (Cat#AF7288) antibody was purchased from R&D Systems. Anti-Sheep IgG HRP-coupled secondary antibody (Cat#A3415), anti-puromycin antibody (12D10, Cat#MABE343), Anti-ZMYND19 antibody produced in rabbit (Cat#HPA020642) and anti-GFP (GF28R, Cat# MA5-15256) antibody were purchased from Millipore Sigma. Anti-MKLN1 (C-12, Cat#sc-398956), anti-ZMYND19 (E-4, Cat#sc-398514) and anti-LAMP1 (H4A3, Cat#sc-20011), anti-BZLF1 (BZ1, Cat#sc-53904), anti-BMRF1 (0261, Cat#sc-58121), anti-EBNA1 (1EB12, Cat#sc-81581) antibodies were purchased from Santa Cruz Biotechnology. WDR26 antibody (Cat#A302-245A) was purchased from Fortis Life Science. Anti-LAMP-2 antibody (Cat#H4B4) was purchased from DSHB. Alexa Fluor® 594 AffiniPure Donkey Anti-Mouse IgG (H+L) (Cat#715-585-150) and Alexa Fluor® 488 AffiniPure Goat Anti-Rabbit IgG (H+L) (Cat#111-545-144) were purchased from Jackson ImmunoResearch.

Validation

The antibodies obtained from commercial sources were validated by the suppliers mentioned earlier. Validation details, including immunoblot, immunofluorescence, and knockout analyses, are provided on the suppliers' websites and in the publications they cite.

Eukaryotic cell lines

Policy information about cell lines and Sex and Gender in Research

Cell line source(s)

The EBV+ gastric cancer cell line YCCEL1 was obtained from Elliott Kieff. The EBV+ cell line SNU-719 was obtained from Adam Bass. The EBV- gastric cancer cell line HGC-27 was obtained from Sigma, SNU-1 and SNU-16 were from ATCC. HEK-293T was obtained from ATCC. P3HR-1 with conditional ZTA and RTA immediate early alleles triggered by 4-hydroxytamoxifen (4-HT) were obtained from Elliott Kieff.

Authentication

YCCEL1 and SNU719 cells were authenticated by the Idexx CellCheck 9 - human STR Profile and Inter-species Contamination Test. Cell lines expressing new constructs (KO or OE) were validated via WB.

Mycoplasma contamination

Cell lines tested negative for mycoplasma.

Commonly misidentified lines
(See ICLAC register)

No commonly misidentified cell lines were used in this study.

Plants

Seed stocks

n/a

Novel plant genotypes

n/a

Authentication

n/a

Flow Cytometry

Plots

Confirm that:

- [x] The axis labels state the marker and fluorochrome used (e.g. CD4-FITC).
- [x] The axis scales are clearly visible. Include numbers along axes only for bottom left plot of group (a 'group' is an analysis of identical markers).
- [x] All plots are contour plots with outliers or pseudocolor plots.
- [x] A numerical value for number of cells or percentage (with statistics) is provided.

Methodology

Sample preparation	For cell cycle analysis, cells were trypsinized, pelleted, washed once with FACS buffer (2% FBS v/v in PBS), fixed in 70% ethanol overnight at 4oC, washed twice with PBS, and treated with staining buffer (propidium iodide 5µg/ml, RNase A 40µg/ml and 0.1% Triton X-100 in PBS) for 30 minutes at room temperature. For glucose uptake analysis, live trypsinized cells were washed once with PBS and incubated with 10 mg/mL 2-NBDG in complete media at 37oC.
Instrument	BD FACSCalibur
Software	Flowjo X
Cell population abundance	Approximately 1 million cells were collected per group.
Gating strategy	For cell cycle analysis, all cells were included. For glucose uptake analysis, all alive cells were included.

- [x] Tick this box to confirm that a figure exemplifying the gating strategy is provided in the Supplementary Information.
